# Transgenerational genetic and epigenetic changes induced by gamma-ray in *Fagopyrum* species

**DOI:** 10.1186/s12870-025-07033-4

**Published:** 2025-07-31

**Authors:** Katarzyna Sala-Cholewa, Magdalena Rojek-Jelonek, Jolanta Kwasniewska

**Affiliations:** Institute of Biology, Biotechnology and Environmental Protection, Faculty of Natural Sciences, University of Silesia in Katowice, Jagiellonska 28, Katowice, 40-032 Poland

**Keywords:** DNA damage, DNA methylation, Fagopyrum, Gamma-ray, Micronuclei, Transgenerational inheritance

## Abstract

**Background:**

DNA changes induced by stress may be stable through cell division and passed to subsequent generations. Plant improvement programs require that individuals develop a new heritable trait. The action of mutagens applied in classical mutagenesis is known from their transgenerational inheritance, observed as mutations. DNA methylation is an epigenetic mechanism that generates heritable phenotypic variation by influencing gene expression and modifying DNA accessibility to mutagens. *Fagopyrum esculentum* (common buckwheat) and *F. tataricum* (Tartary buckwheat) are essential for food production and valuable for medical purposes.

**Results:**

In this study, we aimed to investigate the transgenerational changes in the genome and epigenome of two Fagopyrum species, by analysing 1st and 3rd generations, followed by treatment with gamma rays. The genomic instability, observed as DNA fragmentation, micronuclei, and changes in cell cycle profile, was observed in 1st generation, whereas 3rd generation shows recovery in DNA stability. Gamma-ray stress caused alterations in the pattern and level of DNA methylation in the roots of both analysed generations. The response to gamma rays depends on the species – in 1st generation, a higher increase in DNA methylation level in *F. esculentum* and only slight changes in *F. tataricum* were observed. The DNA methylation level in the 3rd generation changed only in *F. esculentum.*

**Conclusions:**

Gamma ray is a stress factor that affects the pattern and level of DNA methylation in *F. esculentum* and *F. tataricum.* Significant differences were observed between control and 3rd generation only in *F. esculentum.* Differences in DNA methylation following gamma irradiation between the analysed Fagopyrum species may result from various forms of pollination in the analysed species. By using gamma ray-induced mutation, we got a highly stable mutant of *F. tataricum*, which may result in a self-pollinating trait. Understanding the changes in DNA methylation following mutagenic treatment can be essential in a breeding programme.

**Supplementary Information:**

The online version contains supplementary material available at 10.1186/s12870-025-07033-4.

## Background

Plants have evolved modulating mechanisms to respond to various stress conditions. The genomic response plays the most important role, which includes genetic and epigenetic alterations [1]. DNA changes may be stable through cell divisions and passed to subsequent generations [2]. These long-term changes are called the transgenerational inheritance [3]. Heritable DNA changes the environment induces become natural, potentially significant evolutionary forces [4–6]. An induced transgenerational genome instability strongly depends on the stress type [7]. Among different epigenetic modifications, DNA methylation is transgenerationally inheritable in response to various stressful conditions [8]. DNA methylation is a reversible enzyme-mediated DNA modification that controls the activity of genes, repetitive sequences, and transposable elements. DNA methylation is essential for genome integrity [9]. Global DNA methylation is species- and tissue-specific and can also vary in different cells of the organism [10]. Despite the comprehensive studies, the transgenerational inheritance mechanisms remain not fully understood in plants. More studies with different stressors are needed to elucidate the mechanisms of transgenerational heritability of genetic and epigenetic effects.

Plant improvement requires that individuals develop a new trait, important from an agronomical point of view, that is passed from one generation to the next. These traits comprise flowering time, plant height, yield, pests, and environmental resistance [11]. Of all the stress factors, chemical and physical mutagens commonly applied in classical mutagenesis are known from their transgenerational inheritance, observed as mutations. Simultaneously with genetically fixed mutations, DNA methylation is one mechanism that generates novel and heritable phenotypic variation by influencing gene expression [12–14]. Changes in epigenetic marks could result in a ‘genomic shock’ and increase the frequency of mutagenic events [1]. It must be emphasized that epigenetic changes resulting in alterations in chromatin conformation and subsequently, DNA accessibility to mutagens, could influence genome stability. The relationship between epigenetic variability and stable genetic changes is crucial for new traits over multiple generations [6, 15]. Thus, increasing and identifying heritable epigenetic phenotypes could enhance the efficiency of crop breeding programs [14, 16].

Characterizing the nuclear genome integrity and cell cycle profile is key in breeding programs to improve crop yield and biotic and abiotic stress resistance [17]. Genetic variation in mutant lines developed using mutation techniques must be analyzed in breeding programs to characterize plant biodiversity [18–20]. Currently, advances in molecular biology enable the characterization of mutant lines with complex traits in crop improvement [21]. Among them, the cytogenetic techniques allow analysis in a single nucleus and help characterize the nuclear genome [22, 23].

Fagopyrum species (buckwheat) is a rich source of phenolic compounds, such as rutin, quercetin, and vitexin. These compounds protect against free radicals and have a therapeutic and dietary impact on human health [24–26]. Although buckwheat species are considered minor crops, they are very popular among consumers due to their nutritional properties. Most of the Fagopyrum species are wild buckwheat species, but two of them, *Fagopyrum esculentum* (common buckwheat) and *F. tataricum* (Tartary buckwheat), are the most widely cultivated. Both species are characterized by short growth periods (90–100 days), relatively low soil requirements, high sensitivity to frost, drought, and large temperature fluctuations [27]. *F. esculentum* is an obligatory cross-pollinating, heterostylous species with two floral morphs, Pin and Thrum. *F. tataricum* is a self-pollinating, homostylous species eliminating reliance on external pollinators [28, 29].

The general aim of this study was to gain detailed data on the transgenerational changes in the genome and epigenome of *F. esculentum* and *F. tataricum*, followed by irradiation with gamma rays. This study used histological and molecular approaches to analyze the roots of two generations, the first (M1) and third generation (M3), after treatment, together with the control one (C). To analyze the genome instability, the analyses of (i) the cell cycle profile using flow cytometry and mitotic activity index, (ii) DNA breaks with TUNEL assay, and iii) frequency of micronuclei were applied. The qualitative and quantitative analyses of DNA methylation were applied to investigate whether gamma ray affects the epigenomes of *Fagopyrum* species. Regarding this aim, the detailed questions were as follows: (1) whether gamma ray stress may cause alterations in the pattern and level of DNA methylation and (2) whether the level and pattern of DNA methylation after gamma ray treatment were inheritable to the next generations. The quantitative and qualitative analyses of DNA methylation profiles on histological sections, followed by physical mutagens, were applied for the first time.

## Methods

### Plant material and treatment

**Fig. 1:**
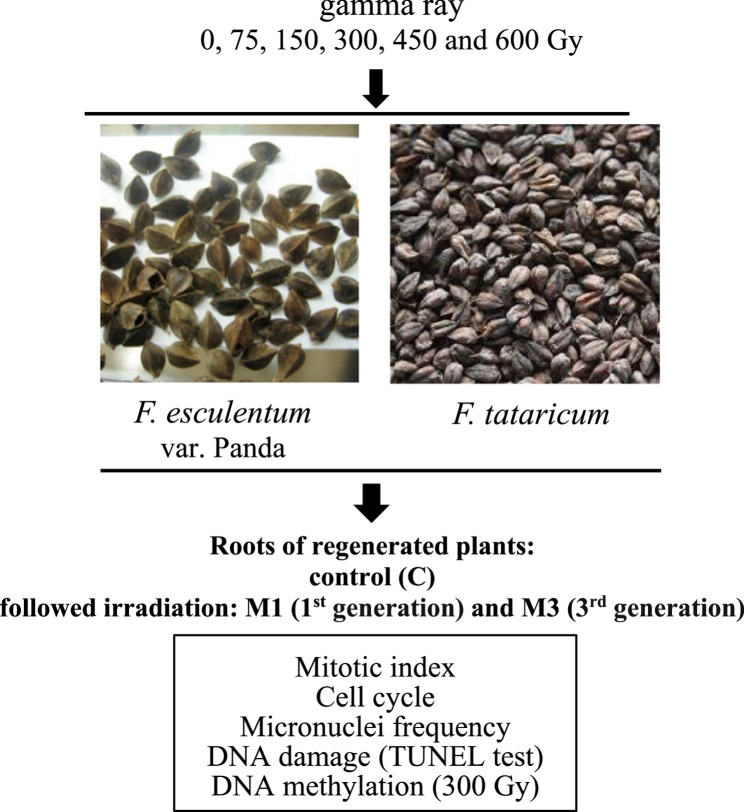
Schematic diagram showing experiments and type of plant material

Seeds of *Fagopyrum esculentum* Moench var. Panda and *F. tataricum* (L.) Gaertn. were used as a source of material. Gamma radiation for seeds was used in the following doses: 0, 75, 150, 300, 450 and 600 Gy. The irradiation was conducted in the International Atomic Energy Agency, Seibersdorf Laboratory, Austria. The *F. esculentum* and *F. tataricum* seeds were sown in the greenhouse at 25 ± 1°C and a 16 h photoperiod with a light intensity of 40 μm/s/m^2^ for obtaining control, 1 st generation followed irradiation (M1) and 3rd generation followed irradiation (M3). The roots of control, M1 and M3 were used for analyses. Gamma irradiation of *F. esculentum* resulted in an increased yield in M3 generation by 26%, and of *F. tataricum* by 19%. The schematic representation of all experiments is shown in Fig. 1.

### Mitotic activity and the frequency of micronuclei

For these analysis, the seeds were presoaked in distilled water for 8 h and germinated in Petri dishes at 21 °C in the dark. Roots were fixed in methanol: acetic acid (3:1 v/v) for 4 h at room temperature (RT) to analyze the mitotic activities and frequency of nuclei with micronuclei. Cytogenetic preparations of root meristems (each made from one plant) were done as described by Perez-Perez and Kwasniewska [30]. Excised root tips were digested for 1.5 h at 37 °C in a mixture of enzymes: 20% (v/v) pectinase (Sigma-Aldrich) and 1% (w/v) cellulase ‘Onozuka RS’ (Serva). One root for each of the ten plants was used for analyses. The mitotic activity and the frequency of interphase cells with micronuclei were analyzed for 1000 root meristematic cells/nuclei for each slide. Statistical analyses were performed using ANOVA (*P* < 0.05) followed by Tukey’s honestly significant difference test (Tukey HSD test, *P* < 0.05).

### TUNEL test

The TUNEL (terminal deoxynucleotidyl transferase-mediated dUTP nick-end labelling) reaction was applied to detect nuclei with DNA fragmentation. Roots were fixed in freshly prepared 4% paraformaldehyde (Fluka) in PBS (phosphate-buffered saline) for 1 h at RT and then washed for 3 × 5 min in PBS. The nuclei preparations were made using squashing meristems in a drop of PBS buffer. After freezing at −70ºC, the slides can be stored at 4ºC for several days. Before the TUNEL reaction, the slides were air-dried and permeabilized by incubating in 0.1% Triton X-100 (Sigma) in 0.1% sodium citrate at 4ºC for 2 min and rinsed in PBS. A slide was treated with a 50 µl of DNase solution (1U/ml) for 30 min at 37ºC in a humid chamber for the positive control. The labelling of DNA fragmentation was carried out with the TUNEL reaction mixture (*in situ* Cell Death Detection Kit, Fluorescein, Roche, cat. no. 11684795910) containing enzyme solution (terminal transferase) and label solution (FITC-labelled nucleotides). 50 µl of the TUNEL reaction mixture (enzyme solution: label solution, 1:9 v/v) was applied to the preparations and incubated in a humid chamber for 1 h at 37ºC in the dark. The preparations were rinsed 3× in PBS, stained with 4,6-diamidyno-2-fenyloindol (2 µg/ml), air dried, and mounted in a Vectashield medium (Vector Laboratories) [31]. One root for each of the five plants was used for analysis. The frequency of FITC-labelled nuclei in the TUNEL test was established based on the study of 2000 cells on each slide (each made from one root meristem). Preparations were examined with a Zeiss Axio Imager.Z.2 wide-field fluorescence microscope equipped with an AxioCam Mrm monochromatic camera. Statistical analyses were performed using ANOVA (*P* < 0.05) followed by Tukey’s honestly significant difference test (Tukey HSD test, *P* < 0.05).

### Cell cycle profile

Approximately 30–50 root meristems of each experimental groups *F. esculentum* and *F. tataricum* were used for analyses. After mechanical root tip fragmentation, using razor blade in nuclei extraction buffer (CyStain^®^ UV Precise P, 05-5002, Sysmex), nuclei suspension was filtered through a 30 μm nylon mesh to remove any large debris and then stained with staining buffer (CyStain^®^ UV Precise P, 05-5002, Sysmex). Samples were incubated for 12 min and analyzed using a CyFlow Space Sysmex flow cytometer with a 365 nm UV LED as the light source. Two samples were analyzed for each experimental group. The results are presented as histograms prepared using a linear scale. The frequencies of cells in particular cell cycle phases, G1, S and G2, are shown. The software FloMax with the Cell Cycle Analysis application was used for cell cycle phase determination.

### DNA methylation - histological and immunostaining procedures

Based on cytogenetic analyses, the 300 Gy dose was chosen for DNA methylation experiments. The dose of 300 Gy of gamma ray was recognized as an optimal as it caused the genotoxic effects, but did not stop mitotic activity in Fagopyrum cells. This dose was the most efficient in both species’ increased yield in M3. Three roots from each examined group (control, M1, and M3) were analyzed. Root tips (5 mm) were fixed in 4% paraformaldehyde (Sigma-Aldrich, USA) in 1× phosphate-buffered saline (PBS), pH 7.2. For fixation, the material was placed in the vacuum desiccator for 2 h (desiccator chamber fused with ATB PGF 56/4B-11R vacuum pump, 230 V, 0,6 A, 1420/min, 50 Hz), and then incubated at 4°C overnight. The next day, the fixative was replaced with 1× PBS (twice for 15 min), and samples were dehydrated in a graded ethanol series diluted in 1× PBS solution (10%, 30%, 50%, 70% and 90% and 99.8% twice for 30 min each). Subsequently, the embedding procedure in Steedman’s wax was performed according to Wolny et al. [32]. 5 μm thick sections were cut using a HYRAX M40 rotary microtome (Zeiss, Oberkochen, Germany) and placed on poly-lysine-coated microscope slides (Epredia, Netherlands). The slides were de-waxed by incubating in 99.8% ethanol three times for 10 min, followed by the rehydration in ethanol/1× PBS solutions: 90%, 50%v/v and, finally, in 1× PBS, for 10 min each. For preliminary histology visualization, sections were stained with 0.05% Toluidine Blue O aqueous solution (TBO, Sigma-Aldrich), rinsed in water, and photographed in an Olympus BX43F microscope equipped with an Olympus XC50 digital camera.

For immunolocalization, samples were treated with 2 N HCl (Sigma-Aldrich, USA) for 45 min to denature DNA [33]. Then, 3 washes in 1× PBS, 5 min each, were applied to neutralize pH of the root sections. Then, the slides were incubated with 5% bovine serum albumin (BSA, Sigma Aldrich, USA) in 1× PBS for 1 h in the humid chamber at RT. Next, the primary antibody (Anti-5-methylcytosine, Abcam, UK; ab73938) diluted in 1% BSA in 1× PBS (1:100) was applied, and slides were incubated at 4°C overnight. After the incubation, the slides were washed thrice in 1× PBS, 5 min each. Subsequently, a secondary antibody (Goat Anti-Mouse IgG, Abcam, UK; ab150113) diluted in 1% BSA in 1× PBS (1:100) was applied, and samples were incubated at 37°C in a humid chamber in the dark for one h. After the incubation, the slides were washed thrice in 1× PBS, and nuclei were counterstained with DAPI, 2.5 g/ml in Vectashield.

### DNA methylation - fluorescence intensity measurements

Longitudinal sections of *F. esculentum* and *F. tataricum* roots, control (C) and gamma ray-treated of the first (M1) and third (M3) generations were analyzed. The examined areas were the root tips, approximately 300 μm from the end of the root cap. To avoid scanning the identical nuclei, images were collected every third slice. Images were captured with the Olympus FV1000 confocal system (Olympus, Poland) equipped with an Olympus IX81 inverted microscope. Fluorescence of Alexa 488 (excitation 488 nm, emission 500–600 nm) was acquired from a 40x UPlanFL N oil-immersion objective lens (NA 1.30), a 50 mW 405 nm diode laser and a 100 mW multi-line argon ion laser (Melles Griot BV, the Netherlands). An axial series of two-dimensional fluorescence images of the optical sections through the nuclei (z-stacks) was collected with the use of two separate photomultipliers (R6357, Hamamatsu, Japan) set to work in the integration mode at a four-µs pixel dwell time and 12-bit signal digitization. Alexa488 fluorescence intensity levels were measured in the ImageJ version 1.53 s software (Wayne Rasband, National Institutes of Health, USA), according to Tomasiak et al. [34]. Images were converted to eight bits and segmented with the threshold value parameter. The fluorescence intensity of Alexa488 was calculated as mean values from the Integrated Density (IntDen) parameter per one nucleus, which depicted the sum of all the pixels within the region of interest. Results are presented in relative units.

For tissue visualization, sections were counterstained with Fluorescent Brightener 28 (Sigma-Aldrich) and photographed in an Eclipse Ni-U microscope equipped with a Nikon Digital DS-Fi1-U3 camera with corresponding software (Nikon, Tokyo, Japan) and a maximum excitation wavelength of 330 nm. To determine if there are statistically significant differences between 1/control, M1, and M3 generations within each species, the Kruskal–Wallis Test was performed; level *P* ≤ 0.05. All calculations were done in the TIBCO Statistica™ v.13 program. Raw data are presented in the Additional file 1. Photographs were cropped, adjusted (brightness, contrast) and arranged into figures in the Corel Draw 2020 program.

## Results

### Mitotic activity

**Fig. 2:**
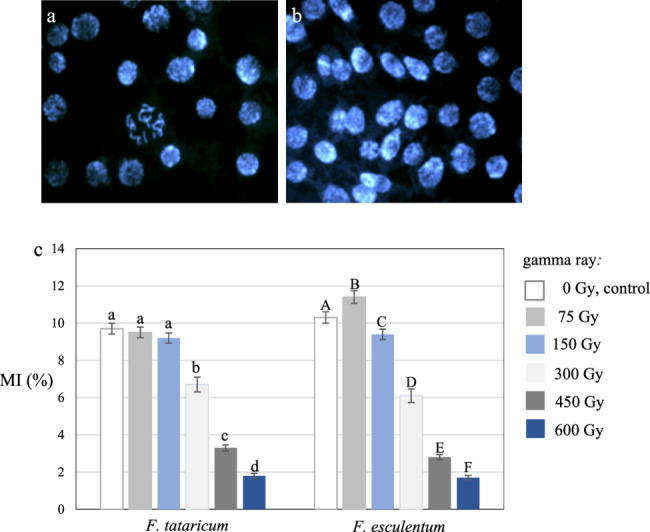
Mitotic index (MI) in the M1, control and gamma ray-treated root meristematic cells of *F. esculentum and F. tataricum*. Examples of control **a** and treated with 300 Gy of gamma ray **b** cells of *F. esculentum*. DAPI staining. Bars represent 10 μm. **c** Mitotic index (%) in control and M1 generation, treated with different doses of gamma-ray, root cells of *F. tataricum* and *F. esculentum* var. Panda. The significant differences (*P* < 0.05) between the groups are indicated by different letters (lowercase letters for *F. tataricum* and uppercase letters for *F. esculentum*).

Treatment with gamma radiation resulted in a tendency to decrease the mitotic index (MI) in M1 roots of *F. tataricum* and *F. esculentum* (Fig. 2). An examples of the cells of *F. esculentum*, control (Fig. 2a) and M1, treated with 300 Gy of gamma ray (Fig. 2b) are shown. The MI in control *F. tataricum* was 9.7% and 10.3% in *F. esculentum* (Fig. 2c). The MI values for M1 roots of *F. tataricum* control and M1 gamma-ray-treated with 75 and 150 Gy showed no significant difference, ranging from 9.2 to 9.7%. The gamma-ray doses of 300, 450 and 600 Gy resulted in significantly lower MI, e.g. the frequency of dividing cells for 600 Gy was only 1.8%. The mitotic activity of M1 roots of *F. esculentum* significantly changed in response to all gamma-ray doses. Surprisingly, a significant increase in MI from 10.3 to 11.4% was observed for 75 Gy gamma-ray-treated roots. M1 roots treated with all other doses of gamma-ray showed significantly decreased mitotic index. The most substantial reduction in the mitotic index was observed for roots treated with 600 Gy, to 1.7%. In contrast to M1, the MI of M3 gamma ray-treated roots of *F. tataricum* and *F. esculentum* were similar to their controls and ranged from 9.5 to 10.3% (data not shown in Fig. 2c).

### Cell cycle profile

**Fig. 3:**
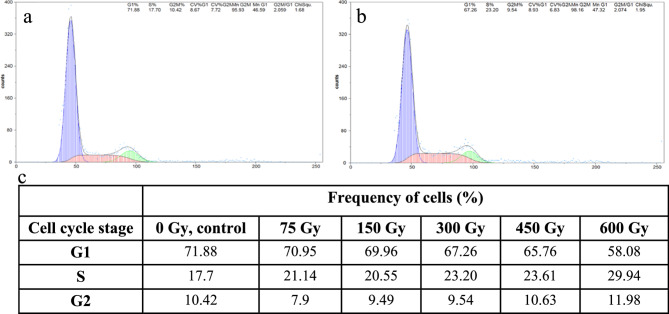
Flow cytometric analyses of cell cycle in M1 roots of *F. tataricum. *Examples of histograms showing cell cycle profiles for control roots **a** and after treatment with 300 Gy of gamma ray **b**, **c** the table summarizing the frequencies of cells in particular cell cycle phases: G1, S and G2

**Fig. 4:**
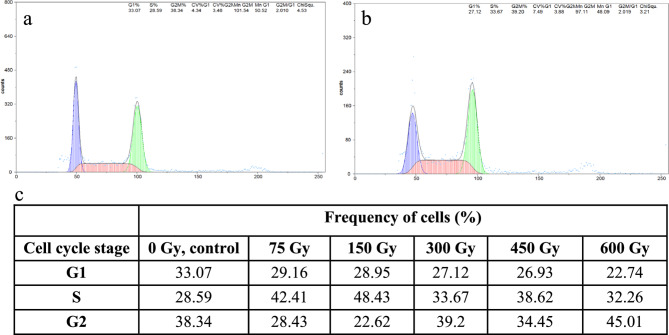
Flow cytometric analyses of cell cycle in M1 roots of *F. esculentum*. Examples of histograms showing cell cycle profiles for control roots **a** and after treatment with 300 Gy of gamma ray **b**, **c** the table summarizing the frequencies of cells in particular cell cycle phases: G1, S and G2

The cell cycle profiles of the control and the gamma-ray treated roots of M1 and M3 generations of *F. tataricum* and *F. esculentum* var. Panda was determined using flow cytometry. The cell cycle profile differed between the control roots of *F. tataricum* and *F. esculentum* (Figs. 3 and 4). Most cells of *F. tataricum* roots were in the G1 phase (71.88%), much less in S (17.7%) and G2 (10.42%). In the roots of control, *F. esculentum*, most of the cells are in G2 (38.34%), then G1 (33.07%) and S phase (28.59%). The tendency to decrease or increase the cell frequencies of G1, S and G2 phases depends on the species. In the M1 roots of *F. tataricum*, there is a dose-dependent tendency to decrease the frequency of G1 cells and increase the frequency of S- and G2-phase cells following the treatment with gamma rays. Different response to gamma rays was observed in the M1 roots of *F. esculentum*. While a decrease in the frequency of G1 cells was observed, no clear tendency was observed for S and G2 cells. The cell cycle profile of M3.

### Micronuclei

**Fig. 5:**
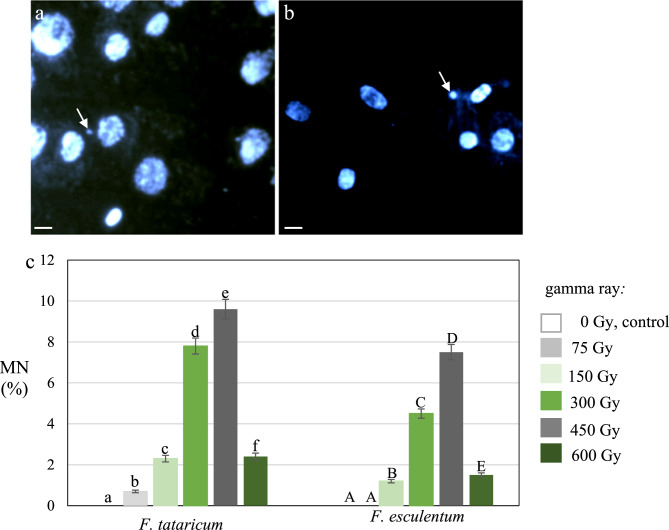
Micronuclei (MN) in the M1 root meristematic cells of *F. tataricum and F. esculentum*. Examples of gamma-ray (300 Gy) treated cells of *F. esculentum ***a** and *F. tataricum ***b**. DAPI staining. Arrowheads point to micronuclei. Bars represent 10 µm. **c**Frequency (%) of MN in the M1 root cells of *F. esculentum *and *F. tataricum, *control and treated with gamma ray. The significant differences (*P* < 0.05) between the groups are indicated by different letters (lowercase letters for *F. tataricum *and uppercase letters for *F. esculentum*)

Analysis of the frequencies of the micronuclei in control and gamma ray-treated M1 and M3 root meristematic cells of *F. tataricum* and *F. esculentum* was done on DAPI stained nuclei preparations (Fig. 5a, b). The results showed the clastogenic effects of gamma ray in M1 roots (Fig. 5c) and no cells with micronuclei in control of both Fagopyrum species. The frequencies of the micronuclei increased with the dose of gamma radiation. However, the highest dose, 600 Gy, caused a significant decrease in the frequency of cells with micronuclei compared with 300 and 450 Gy and similar to dose of 150 Gy in both species. The frequency of cells with micronuclei was higher in *F. tataricum* than in *F. esculentum* in response to the same doses of gamma rays. The highest frequency of cells with micronuclei was observed for 450 Gy, 9.6% and 7.5% in *F. tataricum* and *F. esculentum*, respectively. We did not observe the cells with micronuclei in gama-ray-trated M3 roots (data not shown in Fig. 5).

### DNA damage

**Fig. 6:**
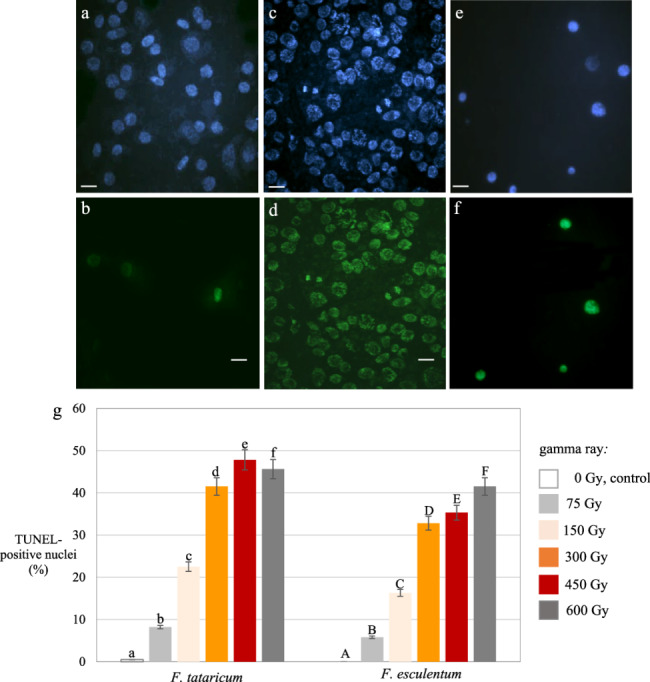
TUNEL test in the M1 root meristematic cells of *F. tataricum *and* F. esculentum*, control and treated with different doses of gamma ray. **a**, **b** Examples of *F. esculentum *nuclei observed in not-treated roots. **c**, **d** Examples of *F. esculentum *nuclei after treatment with DNase–positive control. **e**, **f** Examples of *F. esculentum *damaged nuclei after treatment with gamma ray. **a**, **c**, **e** DAPI-stained nuclei **b**, **d**, **f** FITC fluorescence shows the TUNEL-positive cells. Bars represent 20 µm. **g**Frequency of labelled nuclei in the M1 root cells of *F. tataricum *and* F. esculentum, *control and treated with gamma ray. The significant differences (*P* < 0.05) between the groups are indicated by different letters (lowercase letters for *F. tataricum *and uppercase letters for *F. esculentum *var. Panda)

The TUNEL test was used to detect and analyze the frequency of the nuclei with DNA fragmentation in control (C) and gamma-ray subjected to M1 and M3 root meristems of *F. tataricum* and *F. esculentum*. No TUNEL-specific green-labelled nuclei were observed in the control roots of *F. esculentum* (Fig. 6a, b) and *F. tataricum*. Almost all nuclei showed green fluorescence in the positive control in which a DNase solution induced DNA strand breaks (Fig. 6c, d). A positively stained nuclei were observed following the gamma ray treatment (Fig. 6e, f). A dose-dependent response to gamma-rays as the increased frequency of TUNEL-positive nuclei was observed in both species. However, the higher frequencies of nuclei showing DNA fragmentation in *F. tataricum* than in *F. esculentum*, following the same doses, were observed e.g. 47.8% in *F. tataricum* and 35.3% in *F. esculentum* after 450 Gy (Fig. 6g). No nuclei with DNA damage were detected in M3 roots of both species (data not shown in Fig. 6).

### Anatomy of roots

**Fig. 7:**
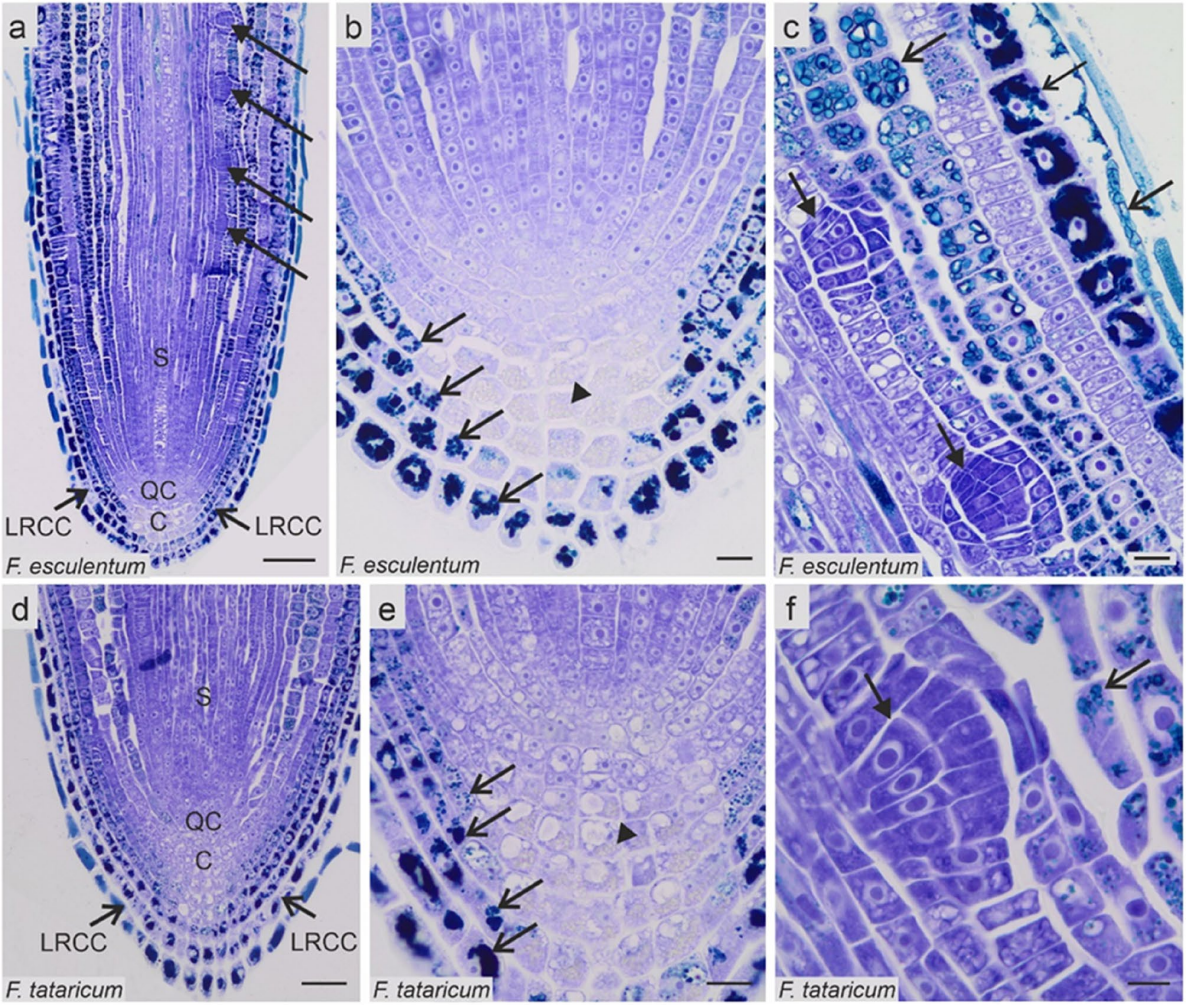
Histology of the *Fagopyrum esculentum *and *F. tataricum *control root tips. Arrows – lateral root primordia, open arrows – phenolic compounds, arrowheads – starch. C columella, LRCC lateral root cap cells, QC quiescent centre, S stele. Scale bar: a = 100 µm; b, c, e = 20 µm, d = 50 µm, f = 10 µm

**Fig. 8:**
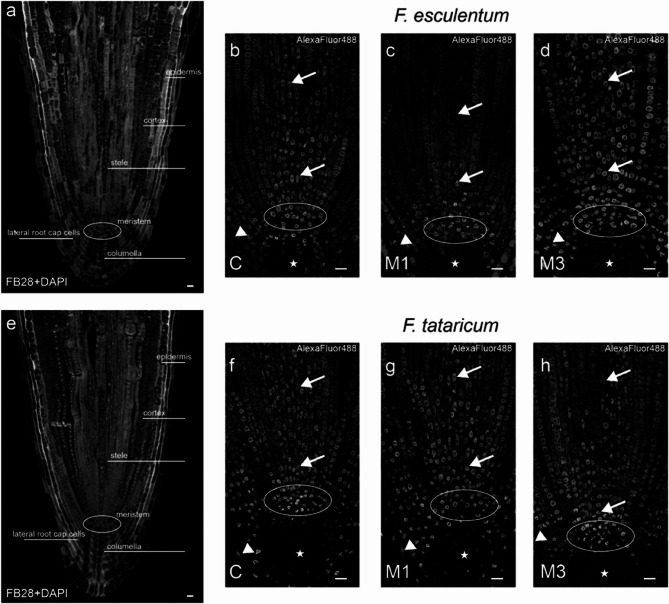
Fluorescence images of the examined root tips. **a**, **e** General visualization of the analyzed root area, sections counterstained with DAPI (nuclei) and Fluorescent brightener 28 (cellulose in cell walls). Alexa 488 fluorescence **b**-**d**; **f**-**h**) in: arrows – stele; circles – meristem; asterisks – columella; arrowheads – lateral root cap cells. Scale bar = 20 µm

Before examining DNA methylation on *Fagopyrum* root sections, we performed histological analyses. Quiescence centre (QC), columella, as well as stele, ground and covering tissues with their initials were distinguished in RAM of *F. esculentum* and F. *tataricum* (Figs. 7 and 8). A starch granules was detected in columella cells (Fig. 7b, e). The root tip was enfolded in 3 layers of lateral root cap cells. It represented typical root anatomy, which comprised the epidermis, three layers of cortex, endodermis, and stele (Fig. 7a-f). Lateral roots developed due to cell divisions in the pericycle (Fig. 7a, c, f). Roots of both species contained large amounts of phenolic compounds, which were accumulated predominantly in root cap cells, epidermis, and cortex (Figs. 7 and 8a-f). No anatomical differences between control roots and M1 and M3 generations following gamma ray treatment were observed. The only difference was the development of lateral roots closer to the QC in M3 gamma-treated roots than control ones (based on visual observation, data not shown in the Figures).

### DNA methylation

**Fig. 9:**
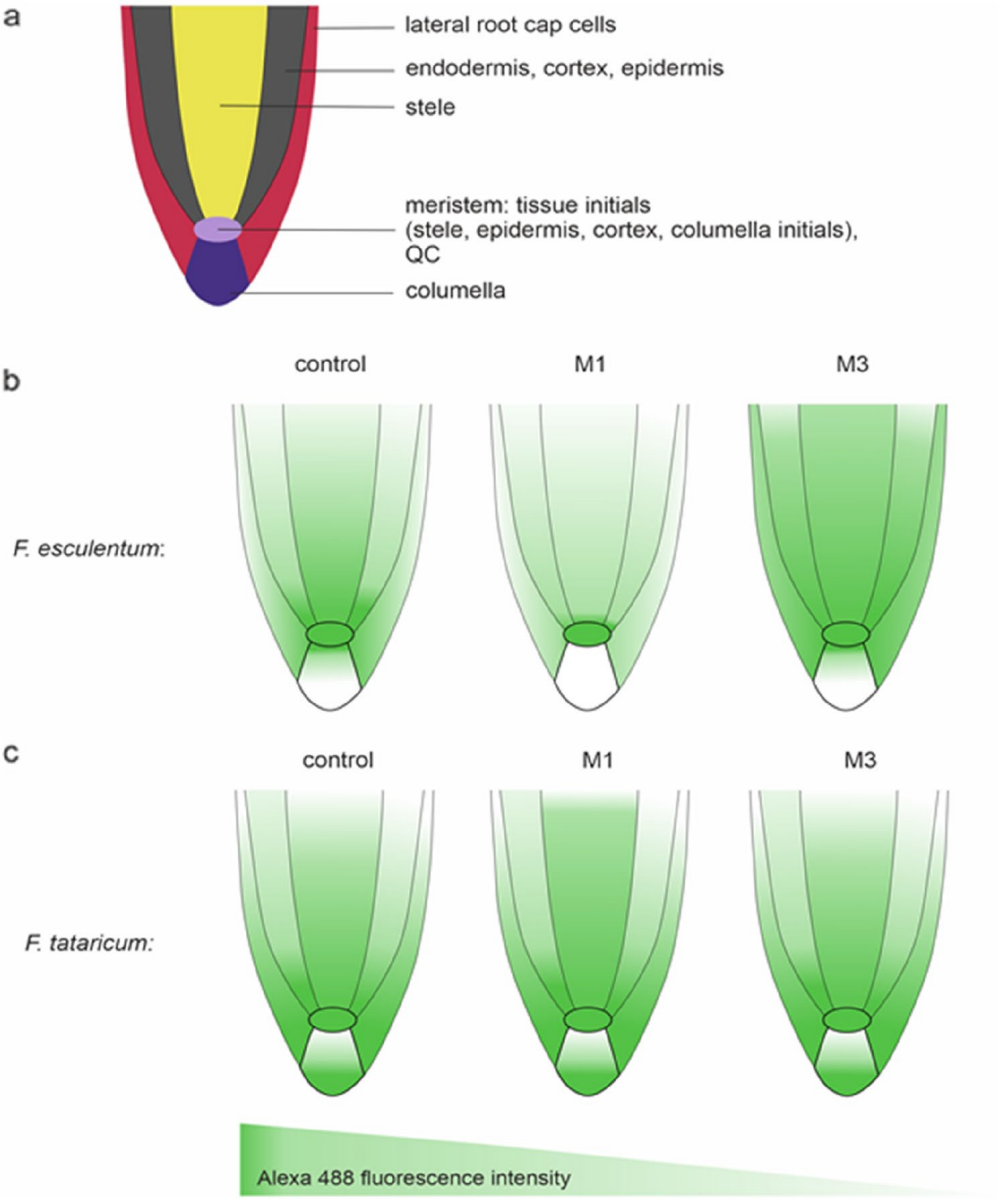
Schematic diagrams representing root histology with Alexa 488 fluorescence intensity distribution.**a** root histology **b**, **c** distribution of Alexa fluorescence intensity in *F. esculentum***b** and *F. tataricum***c** in control and followed gamma ray treatment through generations

A qualitative and quantitative analysis of DNA methylation on control roots (C) and gamma ray-treated M1 and M3 roots of both Fagopyrum species was performed (Figs. 8 and 9). To simplify the description of received data, root apical meristem (RAM) of examined species was divided into sections: 1/meristem (area that included: QC and initials of columella, cortex, epidermis and root cap cells), 2/columella, 3/stele, 4/ground and covering tissue (epidermis, cortex, endodermis), 5/lateral root cap cells.

Visual observations showed that control root tips of *F. esculentum* were characterized by strong Alexa488 fluorescence in the nuclei of the meristem area and some columella cells. The lateral root cap and stele nuclei exhibited weaker fluorescence signals (Fig. 8b). After irradiation, in the M1 generation, Alexa 488, fluorescence was weaker in the stele, columella, and lateral root cap cells. Still, the meristem showed no visible differences regarding the signals compared to the control (Fig. 8c). Surprisingly, the M3 generation, following irradiation, exhibited increased fluorescence intensity in most examined areas of the root tip, stele, meristem, and lateral root cap cells (Fig. 8d).

Control roots of *F. tataricum* displayed strong fluorescence in the meristem area and some lateral root cap cells, whereas no signals were observed in the columella. Stele nuclei exhibited moderate fluorescence (Fig. 8f). Images from the M1 and M3 generations after irradiation showed only slight differences in Alexa 488 fluorescence. Specifically, in M1 roots, the signals were slightly stronger in the stele, while in M3, the fluorescence distribution was similar to that of the control (Fig. 8f-h). A schematic representation of the distribution of fluorescence signals and fluorescence intensity in root tissues, control and treated with gamma rays of *Fagopyrum esculentum* and *F. tataricum* species is shown in Fig. 9.

**Fig. 10:**
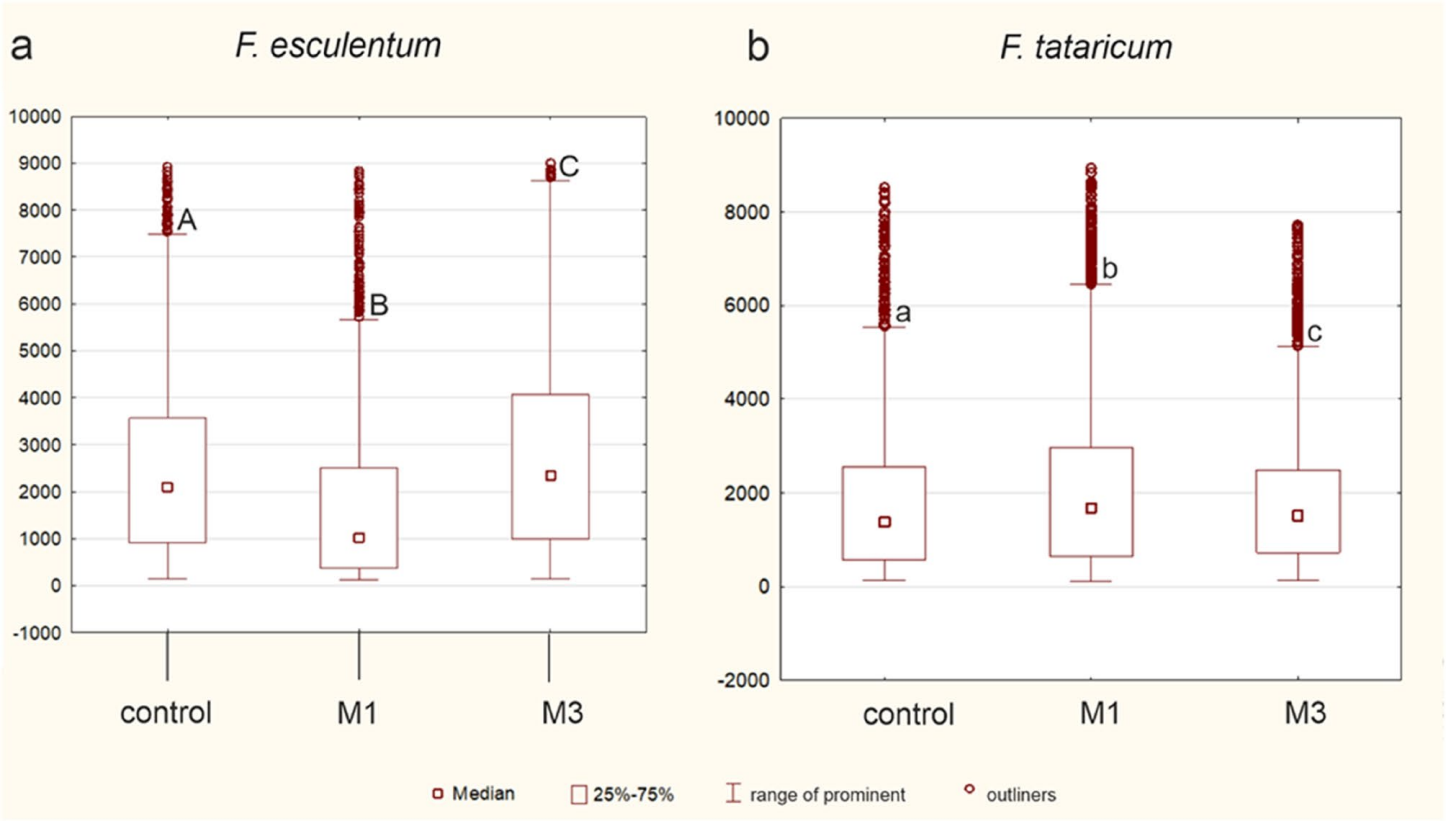
Schematic representation of DNA methylation level in examined root tips. **a** fluctuation of DNA methylation between control, M1 and M3 generations of *F. esculentum*. **b** fluctuation of DNA methylation between control, M1 and M3 generations of *F. tataricum*. The significant differences (*P* < 0.05) between the groups are indicated by different letters (lowercase letters for *F. tataricum *and uppercase letters for *F. esculentum *var. Panda)

The DNA methylation level in control *F. esculentum* roots was around 2700 relative units. The values decreased in the irradiated roots of the M1 generation. In contrast, in the M3 generation, the DNA methylation level was significantly higher compared to control roots (Fig. 10a). The output values of DNA methylation in control roots of *F. tataricum* were at a lower level, around 1800 relative units, than in control *F. esculentum*. The differences in the response of *F. tataricum* compared to *F. esculentum *to gamma-ray roots were observed. Namely, the generation of M1 *F. tataricum* roots exhibited slightly but significantly increased values of DNA methylation, while M3 expressed a value similar to the control mean value (Fig. 10b).

## Discussion

The main objective of this study was to investigate whether epigenetic and genetic changes induced by gamma rays are transgenerationally inherited in *F. esculentum* and *F. tataricum* roots. Following gamma-ray irradiation, we demonstrated the genomic instability, observed as DNA damage, presence of micronuclei, and changes in cell cycle profile in the root meristematic cells of M1 generation. A recovery of DNA stability in the M3 generation means that their transmission from generation to generation is not universal. Nuclear stability of M3 roots meristematic cells, confirmed by lack of DNA damage, may suggest that DNA repair processes are very efficient in Fagopyrum cells. It is possible that meiosis effectively filters changes induced by gamma ray as it happened in other species [35]. However, DNA damage detected in M1 still may lead to mutations, as many mutations originate from DNA damage that is not properly repaired.

Physical mutagens are powerful tools in plant mutation breeding to induce point mutations, but they also induce a spectrum of cytogenetic effects, e.g. chromosome aberrations and direct DNA damage, ranging from mild to severe. The cytogenetic parameters are excellent makers for dose optimization, screening for genomic stability and selection over generations for utilizing beneficial mutations [36]. Assessment of cytological abnormalities is of utmost important as it not only reflects responsiveness of the species to mutagen, but also signifies sub-lethal doses to be monitored for successful mutagenesis experiments. Mutagenic exposure in one generation often leads to chromosomal instability in future generations even if they’re not directly exposed [37]. The formation of cytological changes, such as chromosome rearrangements, followed a wide range of mutagens, is well documented in plants [38, 39]. Genome instability including chromosomal rearrangements is commonly screened in obtained mutant lines with cytogenetic molecular techniques, such as fluorescent in situ hybridization (FISH). Analyses of other cytological markers of genome stability, like direct DNA damage or cell cycle disturbances are not usually carried out in plant generation that is not directly treated with mutagen. An example of comprehensive analyses comprises many cytogenetic endpoints is the study on the late mutant lines of *Phaseolus vulgaris* [20]. Chromosome instability regarding 35 S rDNA, with an unchanged number of chromosomes was revealed in this species. In contrast to our studies, the differences regarding the profiles of cell cycle between the late mutant lines and the initial genotype were revealed. Also, TUNEL assay, applied to analyze whether the presence of nuclei with DNA breaks characterizes these mutant lines, showed a high level of DNA breaks in some of them. In contrast, no DNA damage was detected in M3 generation of Fagopyrum species in our study.

In this study, getting knowledge about the features of the nuclear genome in the M1 generation, followed gamma ray treatment, helped obtaining new, improved genotypes. We preliminary used a few doses: 75, 150, 300, 450, and 600 Gy for early characterization of cytogenetic effects - mitotic index and DNA damage, such as DNA fragmentation and micronuclei. The 300 Gy of gamma ray for further analyses of DNA methylation was chosen based mainly on cytogenetic analyses in M1. 300 Gy of gamma ray significantly decreased the mitotic activity and showed strong genotoxic effect, which was observed as DNA fragmentation and micronuclei in both Fagopyrum species. The most substantial reduction in the mitotic index was observed for roots treated with 450 and 600 Gy. However, after these doses, the reduced plant growth in the M1 generation was observed (data not shown), so we did not choose them to obtain the subsequent generations. An additional reason for choosing a dose of 300 Gy of gamma ray was its efficiency in obtaining the M3 Fagopyrum mutant lines with the increased yield - by 26% in *F. esculentum* and *F. tataricum* by 19%. It should be emphasized that choosing the same dose for both Fagopyrum species made the cross-species comparison of DNA methylation possible.

DNA methylation is a well-studied epigenetic mechanism involved in stress response. It plays a crucial role during treatment, as well as in DNA repair processes. Since hypomethylated loci are more prone to genomic rearrangements [40], a stress-induced increase in DNA methylation or just a high level of DNA methylation may represent a defensive response protecting from genome rearrangements.

Roots have a unique epigenetic landscape compared to shoots or leaves, each cell line has its own characteristic epigenetic pattern [41]. DNA methylation plays an important role in plant response to the stress by changing gene expression to help them and survive, e.g. genes involved in water balance. Understanding how DNA methylation affects root development could be used to modulate the resilience and productivity of crops - by manipulating the DNA methylation patterns it is possible to create the plants that are characterised by higher yield and more tolerant to stresses. In this study, the measuring the distribution of DNA methylation in the root tissues provides essential insights into the epigenetic effects of physical mutagens. We were interested in whether gamma ray may cause alterations in the pattern and level of DNA methylation in *Fagopyrum esculentum* and *F. tataricum* roots. If so, whether these changes are observed in the next generation - M3, that is not directly exposed to treatment. Results show that gamma-rays affect DNA methylation levels. The response observed as changes in DNA methylation to gamma rays in M1 depended on the species - an increase in the level of DNA methylation compared with the control in *F. tataricum* was observed. In contrast, slight changes compared to control were observed in M1 of *F. esculentum*. The level of DNA methylation changed in M3 generation compared with M1– an increase above the level in the control was noticed in *F. esculentum*. The mean DNA methylation level in the M3 generation of *F. tataricum* was the same as in the control.

Other study showed that treatment with chemical mutagen, 5-azacytidine caused DNA hypomethylation and induced traits of dwarfism and early maturity in flax. Interestingly, the plants could inherit these changes for at least three generations [42, 43]. Studies on Arabidopsis plants exposed to gamma radiation showed global DNA methylation levels increased in the first generation. Then it decreased in the second generation, resulting in values like the parent generation [44]. While *F. tataricum* roots showed similarities to this scenario, *F. esculentum* roots exhibited a reversed reaction to gamma ray treatment. It must be underlined that no changes in the level of DNA methylation in control of subsequent generations for both species were observed in this study. A similar level of DNA methylation in C, M1, and M3 generations can indicate that the changes are inheritable in *F. tataricum*. Still, the level of DNA methylation changed through the generations in *F. esculentum*. The type of pollination can be a key regulating factor in these transgenerational processes. *F. esculentum* is an obligatory cross-pollinating species, while *F. tataricum* is a self-pollinating, homostylous species that eliminates reliance on external pollinators [28, 29]. Notably, *F. tataricum* control and M3 roots showed significantly lower DNA methylation values than *F. esculentum* control and M3 roots. Differences in DNA methylation between those two species were also found during flower development [33].

DNA methylation is species-tissue- and cell-specific process [10]. From the point of view of plant development a high DNA methylation level was observed during the vegetative growth in the shoot apical meristem of *Silene latifolia*. Still, the values decreased upon transition to the floral bud [45]. The data on the distribution of the marks of DNA methylation in not well-recognised in the aspect of mutagenesis. We performed the immunolocalization analyses of root sections to show how DNA methylation marks are distributed in roots and if they are tissue-specific. We also analyzed whether this distribution is changing in response to the gamma-ray and whether there are differences between plant generations. The results indicate high level of DNA methylation in the root meristem, which was defined as an area comprising QC and root tissue initials. Strong fluorescence intensity in root meristem was observed in both the Fagopyrum species’, control and gamma ray-treated of the M1 and M3 generations. Meristems are pools of stem cells required for plant growth and development. They are, therefore, essential targets for crop improvement [46]. High DNA methylation within the meristem decreases DNA accessibility to mutagens, protecting the cells from DNA damage and assuring DNA stability [6, 14, 47]. On the other hand, stable DNA methylation levels do not favour variability [1], which is crucial to improving breeding programs’ efficiency [14, 15]. Another cell type that exhibited a strong fluorescence signal were lateral root cap cells in *F. tataricum* C, M1 and, M3 as well M3 of *F. esculentum*. At the same time, the columella nuclei showed relatively moderate or weak fluorescence. These observations are in contrast to a study of Arabidopsis root, in which the genome of the columella root cap cells was found to be the most methylated [48].

DNA methylation is one of the earliest discovered and most studied regulatory mechanisms in epigenetics, and is considered to be a relatively stable, heritable, transgenerational mark, involving a series of biological processes such as temporal and spatial gene expression [49]. The epigenetic marks can be erased during development and transferred to the next generation. The potentially reversible nature of DNA methylation modifications is mediated either by a passive loss of the methyl groups during DNA synthesis or by active demethylation of previously methylated sequences by the DNA glycosylase [50, 51]. Loss of 5-methyl-cytosines can be also independent of the activity of specific enzymes, as the physical factors cause an increased production of reactive oxygen species (ROS). These factors possess endonuclease activity and causes double-strand breaks (DSBs), which may act as an acceptor for the methyl groups, resulting in loss of methylation. Molinier et al. [52] showed that in Arabidopsis subjected to ultraviolet C, a new trait with significantly increased frequencies of somatic homologous recombination of a reporter transgene occurred and persisted in the next generations. Contrary, a study by Pecinka et al. [7] showed that epigenetic mechanisms-mediated transgenerational inheritance is not common in Arabidopsis if other chemical and physical stress treatments, such as heat, freezing, UV-C, UV-B, bleomycin, and salt, were used. To shed more light on the gamma-ray effect on epigenetic changes and trait inheritance, another DNA methylation mark – 6-methyladenine (6 mA) – could be interesting [53, 54].

Gamma-ray is well known factor leading to point mutations and commonly, applied in classical mutagenesis. We demonstrated its action in changing the DNA methylation profile, that is transgenerationally inheritable in *F. esculentum* roots, and can be responsible for changed variability of mutants lines. However, there is direct evidence that DNA methylation influenced the yield. Interest in transgenerational epigenetic inheritance has intensified with increasing knowledge of epigenetic mechanisms regulating gene expression in response to biotic and abiotic stresses [10, 55–58]. The transgenerational transmission of traits not determined by DNA sequence is of great interest from the point of view of traditional mutagenesis. Epigenetic states of plant genes are often stably transmitted through generations. Epigenetic states of plant genes such as *SUPERMAN* in Arabidopsis [59] and maize transposable elements [60, 61] are stably inherited through generations. Most epigenetic and classical mutations are neutral or deleterious, but transgenerational inheritance might be helpful in breeding programs. A study by Molinier [52] showed the progeny of plants exposed to UV-C had an increased frequency of somatic homologous recombination events. This effect was maintained for a few generations without a stress factor. Numerous advantageous changes in phenotype were considered as the consequences of DNA methylation [62–64]. Evidence suggests that DNA methylation can be transmitted with phenotypic changes, such as fruit pigmentation, independently of DNA sequence changes [58–60]. Genetic variation among the advanced mutant lines is critical to successfully developing new varieties [65, 66]. Understanding the link between genome features and plant phenotypic traits is crucial to prove the mutant nature [67, 68]. Until now, only molecular and cytogenetic techniques have been helpful in characterization of mutant genome organisation. Techniques based on epigenetic markers are promising in expanding knowledge of genome diversity in induced mutagenesis and relating these modifications to improved traits. In the near future characteristics of epigenetic modifications can support the selection of beneficial mutations for finding new, improved genotypes.

## Conclusions

We found that gamma ray is a stress factor affecting the pattern and DNA methylation level. We found differences in the response of *F. esculentum* and *F. tataricum* to gamma-rays in the next generations following gamma-ray irradiation. However, only in F. esculentum, significant differences were observed between control and M3 roots. By using gamma ray-induced mutation we got a highly stable mutant of *F. tataricum*, which may result in self-pollinating trait. Differences in DNA methylation following gamma irradiation between the analysed Fagopyrum species may result from various forms of pollination in the analysed species.

The changes in DNA methylation after gamma ray treatment in *F. esculentum* indicate that these processes could be involved in mutagenesis and potentially relevant to phenotypic alterations. There is a need to integrate epigenetic information into population genetic studies. Our data would provide valuable resources to modulate yield productivity for future buckwheat breeding programs successfully.

## Supplementary Information

Additional file 1: Excel table containing raw numerical data for DNA methylation and statistical analysis of the results.

## Data Availability

All data generated or analyzed during this study are included in this published article (and its additional supplementary information files).

## Abbreviations

BSA: Bovine serum albumin
DAPI: 4,6-diamidyno-2-fenyloindol
*MET1*: DNA methyltransferase 1
PBS: 1x phosphate-buffered saline
TBO: Toluidine blue O
TUNEL: Terminal deoxynucleotidyl transferase dUTP nick end labelling
LRCC: Lateral root cap cells
C: Columella
QC: Quiescent centre
S: Stele
